# Ansa cervicalis or ansa hypoglossi? A systematic review^☆^^☆☆^

**DOI:** 10.1016/j.bjorl.2025.101604

**Published:** 2025-05-29

**Authors:** Gustavo Henrique Pereira Boog, Flávio Carneiro Hojaij, Matheus Mychael Mazzaro Conchy, Renan da Silva Bentes, Flávia Emi Akamatsu, Alfredo Luiz Jácomo

**Affiliations:** a Universidade de São Paulo (FMUSP), Faculdade de Medicina, São Paulo, SP, Brazil; b Universidade Federal de Roraima (UFRR), Boa Vista, RR, Brazil

**Keywords:** Anatomy, Cervical plexus, Hypoglossal nerve, Terminology

## Abstract

**Objective:**

To conduct a literature review on the relationship between the ansa cervicalis (or ansa hypoglossi) and the hypoglossal nerve, discussing this disparity in terminology, and determine which of these terms is the most suitable to identify this important nerve structure.

**Methods:**

This is a systematic review of the literature based on studies found through a standardized search performed at the LILACS and MEDLINE/PUBMED databases; classic anatomy textbooks were also consulted.

**Results:**

Forty-five of the 3731 articles found in the scientific research databases were selected and six classic anatomy textbooks were included in the study. Controversies involving the ansa cervicalis were analyzed and the results clearly showed that most textbooks describe its relationship with the hypoglossal nerve as a temporal junction, with no exchange of nerve fibers. The ansa cervicalis contains fibers arising from the cervical cord, and not from the hypoglossal nerve.

**Conclusion:**

The term ansa cervicalis should be chosen to name this loop of nerves. The term ansa hypoglossi is not structurally correct, and should be abandoned in medical practice, the teaching of anatomy, and in scientific publications.

**Level of evidence:**

Level II.

## Highlights

• There is a disparity of terminology in the literature between ansa cervicallis and ansa hypoglossi.
• This systematic review of 3731 articles proved that the term that best suits anatomical reality is “ansa cervicalis”.
• This conclusion is based on the fact that the nerve fibers arise only from the cervical cord.
• The authors also demonstrated that the term “ansa hypoglossi” should be avoided, in scientific research and clinical/surgical practice.

## Introduction

In the study of Anatomy, disparities between anatomical terminology and terms used in clinical or surgical practice are very common. In most cases, there are no problems associated with these differences. However, when a given term causes conceptual errors about the structures involved, correction is needed. In the Head and Neck Surgery specialty, there is much discussion about Ansa Cervicalis (AC) or Ansa Hypoglossi (AH), as both terms are used to refer to the loop of nerves that composes part of the cervical plexus. The relationship of this loop with the Hypoglossal Nerve (HN) and the disparities between the terms used to describe it are the focus of this study, which is justified by the importance of this structure for several surgical procedures.

The original Latin term ansa cervicalis is used to designate the nerve structure that is a motor portion of the cervical plexus and innervates the infrahyoid muscles, which are used in both phonation and deglutition. Although it presents a high level of anatomical variation, it is generally composed of a descending branch (superior root) arising from the first Cervical nerve (C1) (which is connected to the HN) and an ascending branch (inferior root) arising from the second (C2) and third (C3) cervical nerves. It is the junction of these two branches that creates the form of a loop.

The HN, on the other hand, is the 12th pair of Cranial Nerves (CN XII), and arises from the anterior lateral sulcus of the medulla oblongata, exiting the skull through the hypoglossal canal. It also presents a motor function, being responsible for the movements of the tongue.

The study of the relationship between the AC and the HN has generated controversies since the 19th Century, as several terms have been suggested and adopted to describe this structure: ansa cervicalis, ansa hypoglossi, and ansa hypoglossocervicalis. This study aimed to conduct a literature review on this relationship, which hinders clinical/surgical practice, discussing this disparity in terminology, and to determine which of these terms is the most suitable to identify this important nerve structure.

## Methods

A search using the following standard descriptors was conducted at the LILACS and PUBMED/MEDLINE databases: hypoglossal nerve; hypoglossal nerve OR ansa cervicalis; hypoglossal nerve OR ansa hypoglossi. No time interval was established for the selection of the articles.

Clinical trials, randomized clinical trials, and case reports were excluded from the search. The content selected for this systematic review comprised studies in English, Portuguese, and Spanish with the following characteristics: original research, systematic review and meta-analysis, and retrospective, multicenter and observational studies. In addition, classic textbooks^1^, ^2^, ^3^, ^4^, ^5^, ^6^ of Descriptive and Topographic Anatomy were consulted in their recent editions in English and Portuguese.

## Results

**Fig. 1:**
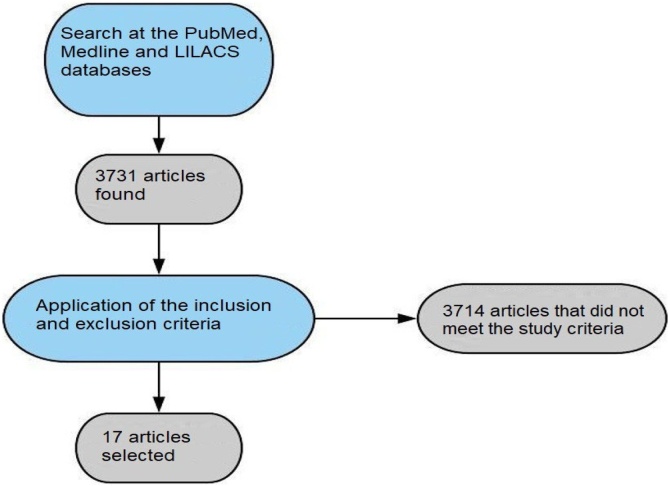
Flow chart of the steps followed to select the articles for the present systematic review of the literature.

Fig. 1 illustrates the results of this search and the selection of articles for the present systematic review.

### Classic anatomy textbooks

In major Anatomy textbooks, identification of the structure in question is practically unanimous, although its description ‒ especially regarding its relationship to the HN - varies largely. Ansa cervicalis is the preferred term in the latest editions of these classic textbooks. This unanimity is in agreement with the International Anatomical Terminology, which uses this term to name this loop of nerves.

Frank H. Netter (Atlas of Human Anatomy)^1^ shows the superior and inferior roots of the AC, evidencing a union of part of the superior root with the HN. Only AC fibers that supply the geniohyoid and thyrohyoid muscles remain joined to the HN. The same situation is described by Michael Shünke (Prometheus Atlas of Anatomy: neck and internal organs),^2^ who states that there is no fiber exchange in the junction of HN and AC. In turn, Friedrich Paulsen (Sobotta Atlas of Human Anatomy)^3^ makes a visual description identical to the aforementioned, and does not mention of any fiber exchange between the two nerve structures in question. Both Prometheus and Sobotta use the term “deep ansa cervicalis” to distinguish the anastomosis between the transverse cervical nerve and the cervical branch of the facial nerve, which composes another neck loop named the superficial ansa cervicalis. Susan Strandring (Gray’s Anatomy: The Anatomical Basis of Clinical Practice)^4^ uses the term ansa cervicalis, but mentions, in parentheses, the term ansa hypoglossi. However, it is specified that the AC superior root contains only fibers arising from C1, and not from the HN, whence it arises. W. Henry Hollinshead (Anatomy for Surgeons: The Head and Neck)^5^ describes the junction between the HN and the superior root of the loop, naming it ansa cervicalis and, like Strandring, mentions the term ansa hypoglossi in parentheses. Hollinshead mentions, also in parentheses, the term “descendens hypoglossi” as an alternative for the superior root of the loop. However, Keith L. Moore (Moore’s Clinically Oriented Anatomy)^6^ does not mention the term ansa hypoglossi, and describes that the superior root of the AC joins momentarily with the HN, but it does not state that this root carries fibers from this nerve (Table 1).

**Table 1:** Summarizes the results of the search conducted in these classic textbooks.

Author	Date of publication	Term used	Relationship with the hypoglossal nerve
W. Henry Hollinshead	1982	Ansa cervicalis, with Ansa hypoglossi in parentheses	Junction, but shows no evidence of fiber exchange
Michael Shunke	2007	Deep ansa cervicalis	Junction, shows evidence of no fiber exchange
Friedrich Paulsen	2012	Deep ansa cervicalis	Junction, but shows no evidence of fiber exchange
Keith L. Moore	2014	Ansa cervicalis	Junction, but shows no evidence of fiber exchange
Frank H. Netter	2015	Ansa cervicalis	Junction, shows evidence of no fiber exchange
Susan Standring	2015	Ansa cervicalis, with Ansa hypoglossi in parentheses	Junction, shows evidence of no fiber exchange

### Scientific research databases

A methodical search conducted at the aforementioned scientific research databases found a total of 3731 articles, of which 45 met the inclusion and exclusion criteria defined for this systematic review. Among these, 17 studies were available and complete (Fig. 1). The most pertinent considerations from the included articles are presented ahead.

The first mention dates from 1825, when French anatomist Jules Cloquet^7^ referred to both branches of the cervical plexus as “ramus descendens cervicalis” and “ramus descendens hypoglossi”, but did not name these branches as loop or ansa. Ten years later, Christophorus Ernestus Bach^8^ presented a doctoral dissertation in which he stated that all AC anastomoses came from the cervical plexus. This was the first time the term ansa cervicalis was used.

Between the late 19th and early 20th centuries, divergent opinions arose about the origin of the AC: Would the nerve branches arise exclusively from the cervical plexus, or would there be a contribution of the HN through anastomoses?

Among the terms that emerged, “ansa hypoglossi” was the most adopted. In 1955, at the VI Federative International Congress of Anatomy in Paris, “ansa cervicalis” was suggested as a universal term and, in 1998, it was presented as definitive terminology.^9^, ^10^ However, disagreements over the ideal terminology and the origin of nerve fibers persist to date.

Several researchers have claimed that only C1 fibers communicate with the HN. This implies the presence of C1 only in the anastomosis with NH and in the three branches arising from this anastomosis (the superior root of the loop and the nerve branches to the thyrohyoid and geniohyoid muscles).^11^, ^12^, ^13^, ^14^ However, morphological studies, such as those undertaken by Kikuchi^15^ and Banneheka,^16^ showed a considerable number of ascending cervical fibers contributing to the superior root of the AC. The difference between these two literatures can be explained by the fact that the studies conducted by Kikuchi and Banneheka analyzed the AC and HN from the microscopic point of view, which explains the same observation by both authors of persisting cervical nerve fibers even after this triple ramification. The findings of these two authors show that cervical nerve fibers join the HN permanently for a short distance. The anatomical consequence of this is that the AC contributes to the HN, but the opposite does not occur. This implication justifies the more descriptive term used by these two authors: “ansa hypoglosso-cervicalis”.

In a study carried out on fetuses using microscopic dissection, Pillay et al.^17^ reported that the superior root of the AC arises from the HN, and not from the cervical cord, as found in previous dissections; however, they did not use the term ansa hypoglossi.

Other published studies using ansa hypoglossi, and not AC, to refer to the structure in question in this review were also found (Table 2).^18^, ^19^, ^20^

**Table 2:** Summarizes this part of the results.

Author	Date of publication	Term used	Relationship with the hypoglossal nerve
Jules Cloquet	1825	None	Does not report anastomosis
Christophorus Ernestus Bach	1835	Ansa cervicalis	Does not report anastomosis
VI Federal International Congress of Anatomy - Paris	1955	Ansa cervicalis	Does not report anastomosis
Kikuchi	1970	Ansa hypoglossi cervicalis	Anastomosis with contribution of ansa cervicalis to hypoglossal nerve
Banneheka	2008	Ansa hypoglossi cervicalis	Anastomosis with contribution of ansa cervicalis to hypoglossal nerve
Pillary	2012	Ansa cervicalis	Superior root of the loop arising from the hypoglossal nerve and not from the cervical plexus

## Discussion

As demonstrated in the results, the term “ansa cervicalis” is used more often than “ansa hypoglossi”, which is in agreement with how this cervical plexus nerve structure is officially named by the International Anatomical Terminology.

However, it is worth discussing the reasons that led to such indiscriminate use of the term ansa hypoglossi. With this regard, reasons can be found in both historical (ansa hypoglossi was a term adopted through common use) and structural (the cervical fibers responsible for the superior root of the loop actually join the HN in a short stretch and separate afterwards) issues. However, this momentary junction does not justify the term ansa hypoglossi; after all, in this junction the fibers arising from the hypoglossal nucleus run parallel to, and independent from, those arising from the cervical cord. Sources mentioning some exchange of fibers between the two nerve structures are actually few, so that most studies do not suggest contribution of the HN to this loop of nerves.

Controversies over this terminology were discussed and, based on the literature revised and compiled in this systematic review, the most appropriate term to meet our initial objective was established. The term that best suits anatomical reality is “ansa cervicalis”, since the nerve fibers arise only from the cervical cord. Thus, we have demonstrated that the term “ansa hypoglossi” should be avoided, both in scientific research and clinical/surgical practice, to minimize the disparities and errors that may arise from the non-universalization of the use of descriptive terms.

## Funding

None.

## Declaration of competing interest

The authors declare no conflicts of interest.

## References

[1] Netter F.H. (2015).

[2] Schunke M., Schulte E., Schumacher U. (2007).

[3] Paulsen F., Waschke J. (2012).

[4] Standring S. (2015).

[5] Hollinshead W.H. (1982).

[6] Moore K.L., Dalley A.F., Agur A.M.R. (2014).

[7] Cloquet J.H. (1825).

[8] Bach C.E. (1835).

[9] Donath T. (1960).

[10] Federative Committee on Anatomical Terminology (1998). Terminologia Anatomica: International Anatomical Terminology.

[11] Iaconetta G., Solari D., Villa A. (2018). The hypoglossal nerve: anatomical study of its entire course. World Neurosurg..

[12] Hall-Craggs E.C.B. (1990).

[13] Caliot P., Dumont D., Bousquet V., Midy D. (1986). A note on the anastomoses between the hypoglossal nerve and the cervical plexus. Surg Radiol Anat..

[14] Sinnatamby C.S. (1999).

[15] Kikuchi T. (1970). A contribution to the morphology of the ansa cervicalis and the phrenic nerve. Acta Anat Nippon..

[16] Banneheka S. (2008). Anatomy of the ansa cervicalis: nerve fiber analysis. Anat Sci Int..

[17] Pillay P., Partab P., Lazarus L., Satyapal K.S. (2012). The ansa cervicalis in fetuses. Int J Morphol..

[18] Sakamoto Y. (2019). Morphological features of the branching patterno of the hypoglossal nerve. Anat Rec (Hoboken)..

[19] Banneheka S., Tokita K., Kumaki K. (2008). Nerve fiber analysis of ansa cervicalis-vagus communications. Anat Sci Int..

[20] Salame K., Masharawi Y., Rochkind S., Arensburg B. (2006). Surgical anatomy of the cervical segment of the hypoglossal nerve. Clin Anat..

